# Some Beneficial Effects of Inert Gases on Blood Oxidative Metabolism: *In Vivo* Study

**DOI:** 10.1155/2022/5857979

**Published:** 2022-12-17

**Authors:** Andrew Martusevich, Alexandra Surovegina, Alexandra Popovicheva, Natalia Didenko, Mikhail Artamonov, Vladimir Nazarov

**Affiliations:** ^1^Laboratory of Translational Free Radical Biomedicine, Sechenov University, 119991 Moscow, Russia; ^2^Laboratory of Medical Biophysics, Privolzhsky Research Medical University, 603005 Nizhny Novgorod, Russia; ^3^MJA Research and Development Inc., East Stroudsburg, PA 18301, USA

## Abstract

The aim of the study was to assess the effect of external use of inert gases (helium and argon) on the state of free radical processes in vivo. The experiment was performed on 30 male Wistar stock rats (age-3 months, weight–200-220 g.), randomly distributed into 3 equal groups. The first group of animals was intact (*n* = 10). The animals of the second and third groups were treated with argon and helium streams, respectively. Our research has allowed us to establish that the studied inert gases have a modulating effect on the state of oxidative metabolism of rat blood, and the nature of this effect is directly determined by the type of gas. The results of this study allowed us to establish the potential antioxidant effect of the helium stream, mainly realized due to the activation of the catalytic properties of the enzymatic link of the antioxidant system of rat blood plasma. At the same time, the revealed features of shifts in oxidative metabolism during treatment with argon flow include not only stimulation of the antioxidant system but also the pronounced induction of free radical oxidation. Thus, the conducted studies made it possible to verify the specificity of the response of the oxidative metabolism of blood plasma to the use of inert gases, depending on their type.

## 1. Introduction

It is an axiom that inert gases under standard conditions do not have the ability to react with other compounds. This circumstance a priori suggests the absence of significant biological and, consequently, sanogenetic effects in them. At the same time, back in the 70s of the last century, isolated works were published on the specific reaction of the body to inhalation of noble gases [1]. In the future, the number of such studies began to gradually increase [2], and in the last decade, there has been a significant increase in interest in the therapeutic potential of inert gases [3, 4]. The spectrum of gases under study includes helium, argon, and xenon [2, 4]. It has been shown that helium and xenon under certain dosage regimens (most often in the form of inhalations of a gas-oxygen mixture in a ratio of 70/30) exhibit neuroprotective [2, 3, 5] and cardioprotective effects [4, 6–10] and are also able to positively affect the functional state of the kidneys [10]. Some publications present hypotheses about the molecular mechanisms of favorable such effects [2, 4, 5, 7, 11]; however, a comprehensive analysis of the problem is necessary to form a full-fledged concept. In particular, considering the data on free radical processes as a universal “biosensor” of various physical and chemical influences [2, 7, 12–14], it is of interest to assess the state of redox processes in biological systems under the action of inert gases, but these data are not available in the literature.

In connection with all of the above, the aim of the study was to assess the effect of the external use of inert gases (helium and argon) on the state of free radical processes in vivo.

## 2. Material and Methods

### 2.1. Experimental Animals and Group Formation

The experiment was performed on 30 male Wistar stock rats (age-3 months, weight–200-220 g). Rats were kept in standard vivarium conditions in cages with free access to food and water on a diet, according to the standards of Russian state standard “Maintenance of experimental animals in Research Institute nurseries.” The animals were obtained from the “Stolbovaya” branch of the Federal State Budgetary Institution of Science “Scientific Center for Biomedical Technologies of the Federal Medical and Biological Agency” (Moscow).

Working with animals was in accordance with the rules of the European Convention ET/S 129, 1986 and Directives 86/609 ESC. The protocol of the study was approved by the Local Ethics Committee of the Federal State Educational Institution “PIMU” (N2/2 from 07/02/2022).

Rats were randomly distributed into 3 equal groups. The first group of animals was intact (*n* = 10). No manipulations were carried out with them, except for a single receipt of blood. The animals of the second and third groups (10 rats each) were treated with argon and helium streams, respectively.

### 2.2. Exposure Procedures

**Figure 1:**
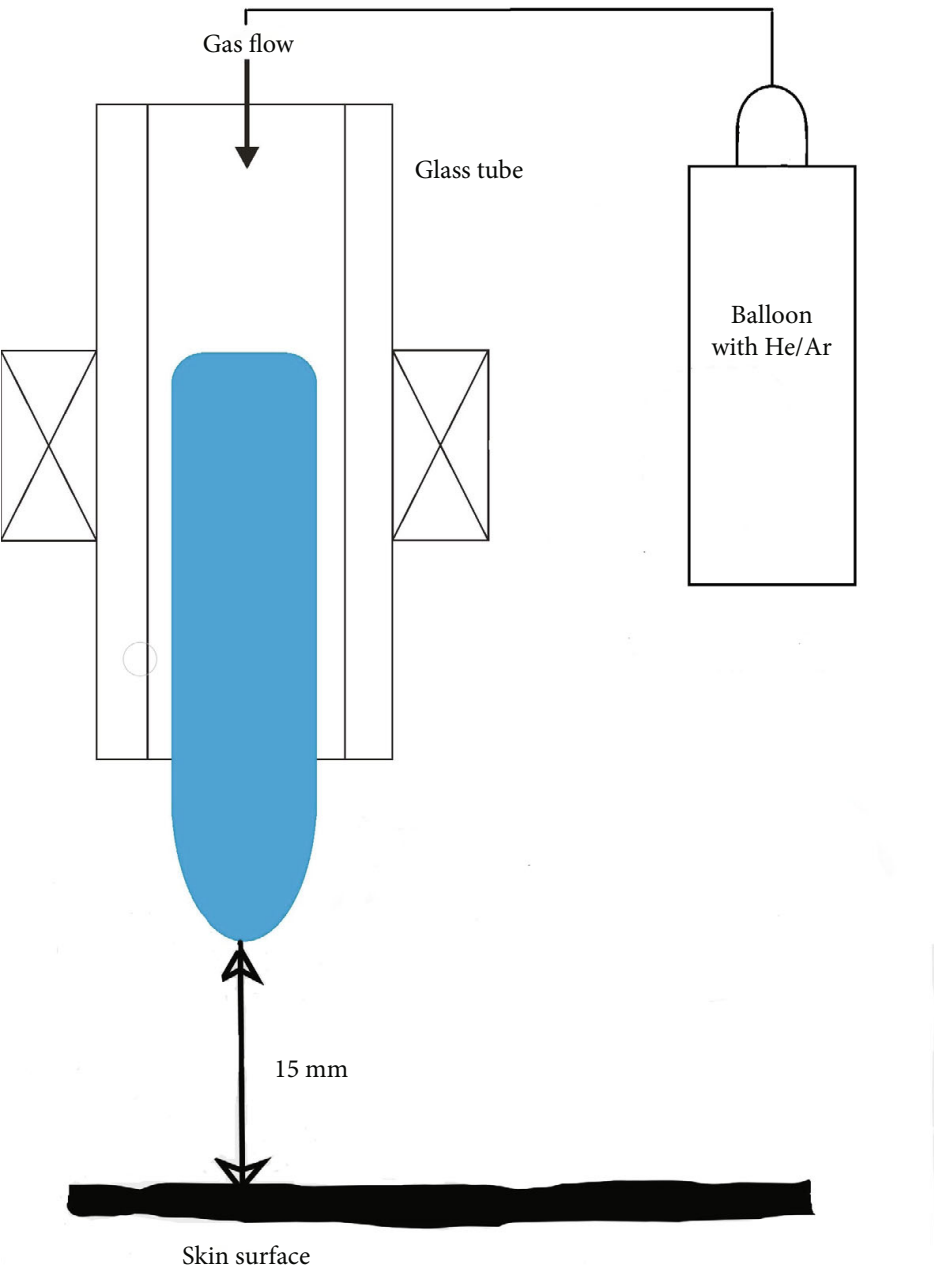
Scheme of experiment performing (He: Helium; Ar: argon).

The treatment was carried out using a special device that provides a gas flow (argon or helium) at a constant speed (Figure 1). This speed was determined by the characteristics of the device and was 1 l/min. The distance from the glass manipulator releasing the gas flow to the surface of the animal's skin was strictly fixed. It was 15 mm. The duration of each procedure was 1 minute, and the course included 3 daily procedures. The effect was performed on identical, premarked, and epilated areas of the middle part of the back.

### 2.3. Laboratory Study

At the end of the experiment, blood samples were obtained from the sublingual vein in all animals (as standard operational procedure), from which plasma was isolated by centrifugation according to the standard procedure (at 1500 g for 10 minutes on ROTOFIX 32A centrifuge, Germany). It evaluated a number of parameters of oxidative metabolism: the concentration of malonic dialdehyde (MDA), the intensity of free radical processes, and the overall antioxidant activity. The level of malonic dialdehyde (MDA) in the blood plasma of animals was assessed using a standard test kit (CJSC AGAT, St. Petersburg). The intensity of peroxidation in blood plasma was studied on the BHL-06 apparatus (Nizhny Novgorod, Russia) by Fe-induced biochemiluminescence according to the maximum flash level, and the total antioxidant activity was studied by the parameter inverse to the chemiluminescence light sum [15].

### 2.4. Statistical Analysis

The results were processed using the Statistica 6.1 for Windows program. The normality of the distribution of parameter values was evaluated using the Shapiro-Wilk criterion. Taking into account the nature of the distribution of the trait, the Kruskal-Wallis *H*-test was used to assess the statistical significance of the differences. Post hoc analysis was carried out using the Bonferroni correction. The differences were considered significant at a significance level of *p* < 0.05.

## 3. Results

**Figure 2:**
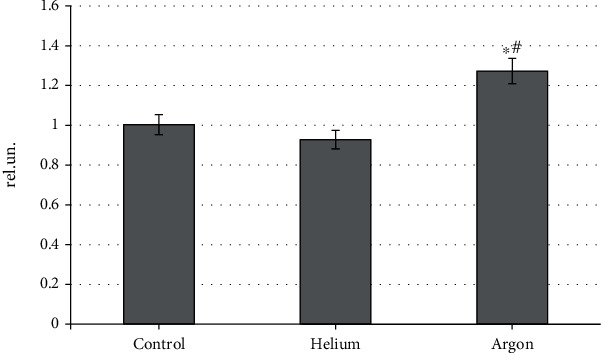
The intensity of free radical oxidation in rat blood plasma when treated with various inert gases (“^∗^”-differences relative to intact animals are statistically significant, *p* < 0.05; “#”-differences relative to intact animals are statistically significant, *p* < 0.05).

Our research has allowed us to establish that the studied inert gases have a modulating effect on the state of oxidative metabolism of rat blood, and the nature of this effect is directly determined by the type of gas. Thus, the noble gases under consideration have an unequal effect on the level of the maximum flash of iron-induced chemiluminescence reflecting the intensity of free radical reactions (Figure 2). It was found that the treatment of animals with a helium stream moderately reduces this parameter (by 8.1%; *p* < 0.05 relative to the control group), while the use of argon increases it by 26.9% (*p* < 0.05). In addition, the level of maximum flash of blood plasma samples of rats treated with argon was statistically significantly higher than that typical for helium (by 37.2%; *p* < 0.05). This may indirectly indicate the prooxidant properties of argon and the antioxidant properties of helium. In our opinion, such a difference in the effect of argon and helium on the oxidative processes of the body is due to the difference in the intracellular signaling pathways involved by them. The involvement of individual molecular cascades by various inert gases is revealed by us based on the analysis of their effect on the components of the antioxidant system of the blood, in particular, on the activity of the enzyme link of this system.

**Figure 3:**
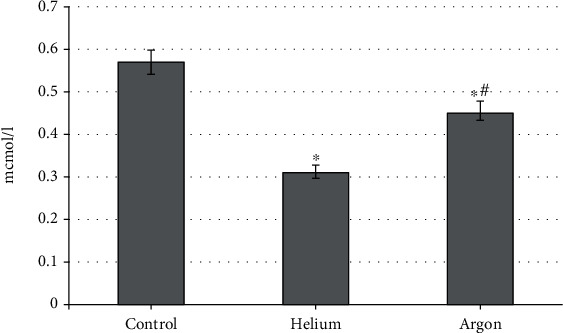
The level of total antioxidant activity of rat blood plasma when treated with various inert gases (“^∗^”-differences relative to intact animals are statistically significant, *p* < 0.05).

The state of the antioxidant potential of animal blood plasma when using both inert gases demonstrates unidirectional trends. It was found that the helium and argon fluxes cause an increase in the corresponding indicator, the inverse of the chemiluminescence light sum (Figure 3). At the same time, the treatment of animals with a helium stream contributes more to an increase in the total antioxidant activity of blood plasma than when using argon (by 47.5% vs. 27.1% relative to the control group; *p* < 0.05 for both effects). This fact, together with the results of the evaluation of the maximum flash, confirms the antioxidant properties of the helium stream.

**Figure 4:**
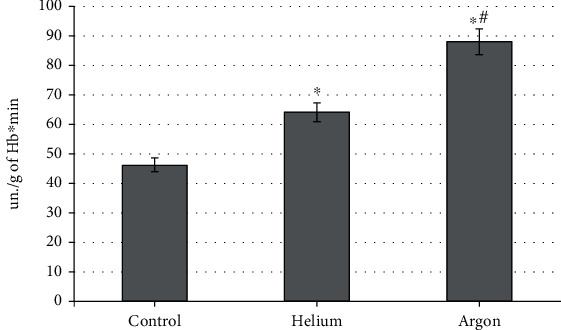
The concentration of malonic dialdehyde in the blood plasma of rats when treated with various inert gases (“^∗^”-differences relative to intact animals are statistically significant, *p* < 0.05).

These shifts in the intensity of free radical oxidation, which occur under the influence of the studied inert gases, are naturally reflected in changes in the concentration of MDA in the blood plasma of rats. It was found that both gases contribute to its reduction, but the severity of this effect is not the same (Figure 4). Thus, the use of helium flux reduces the level of MDA to a greater extent (by 45.4%; *p* < 0.05 compared with the indicator characteristic of intact animals), while the treatment of rats with argon reduces the value of the parameter by 21.1%, respectively (*p* < 0.05).

**Figure 5:**
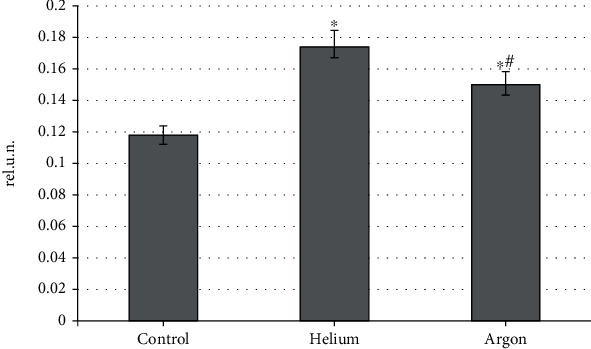
Blood catalase activity during treatment with various inert gases (“^∗^”-differences relative to intact animals are statistically significant, *p* < 0.05).

To reveal the molecular mechanisms of the specificity of the action of the gases under consideration, we evaluated the dynamics of the catalytic activity of the main antioxidant enzymes. It has been found that the influence of the factors under consideration is also different for catalase, which recycles hydrogen peroxide but is codirected (Figure 5). Thus, the treatment of the skin of the animals' backs with a stream of helium contributed to a moderate increase in catalase activity (by 38.9% relative to the control group; *p* < 0.05), whereas the use of argon increased this indicator by 90.7% compared to the level characteristic of intact rats (*p* < 0.01). This indicates a more pronounced increase in the concentration of hydrogen peroxide in the case of argon.

**Figure 6:**
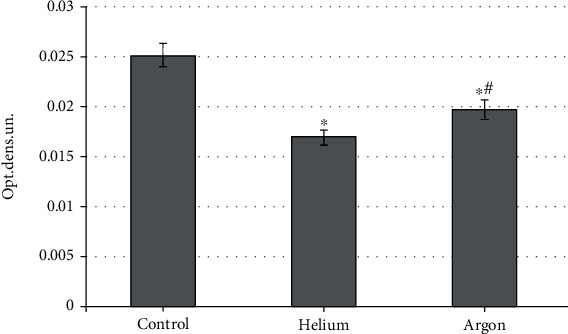
The activity of rat blood superoxide dismutase when treated with various inert gases (“^∗^”-differences relative to intact animals are statistically significant, *p* < 0.05; NADH: reduced form of nicotinamide adenine dinucleotide).

An increase in catalytic activity was also recorded for superoxide dismutase (Figure 6). It is shown that both gases induce an increase in this indicator. It should be noted that, as with catalase, this trend is more pronounced when treating rats with a helium stream than when using argon (+54.6% vs. 29.4% relative to the control group; *p* < 0.05 for both cases). The differences between the groups of animals treated with helium and argon were also statistically significant (*p* < 0.05).

A significant increase in the activity of catalase in the case of argon treatment, and superoxide dismutase in the case of helium treatment, gives us the right to assume that the influence of various inert gases can initiate different ways of implementing antioxidant protection of the body. We assume that the specificity of the response of the antioxidant system of animal blood to the action of the studied inert gases is related to the peculiarities of the molecular products of their interaction with a biological object. The predominant activation of catalase, characteristic of argon treatment, indicates a predominant increase in the concentration of hydrogen peroxide under these conditions. On the contrary, the use of helium promotes the stimulation of the catalytic properties of superoxide dismutase, which indicates the predominant generation of a superoxide radical as a product of the interaction of this inert gas with epithelial cells. At the moment, the question of the optimality of each of the molecular response pathways remains debatable, and only the abovementioned specificity can be fixed.

**Figure 7:**
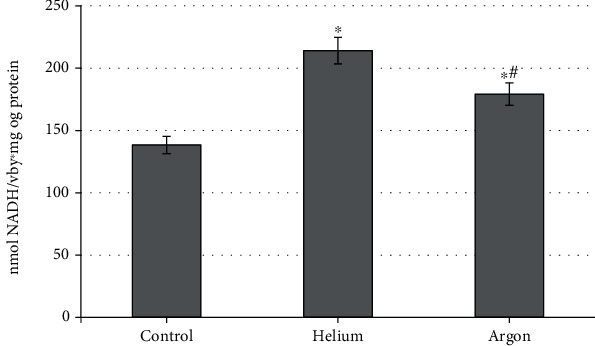
The concentration of ischemically modified albumin in the blood plasma of rats when treated with various inert gases (“^∗^”-differences relative to intact animals are statistically significant, *p* < 0.05).

The presence of antioxidant properties in the studied gases can influence the course of free radical processes in vivo, including oxidative modification of biomolecules. These effects were assessed by the level of ischemically modified albumin (Figure 7). It was found that both gases provide a decrease in the concentration of this compound, and this trend is maximally realized with the use of helium (-32.3% relative to the control group; *p* < 0.05), which has the characteristics of an antioxidant to a greater extent than argon (a decrease of only 21.5%, respectively; *p* < 0.05). It is important to emphasize that the level of the parameter in the treatment of animals with argon is fixed at significantly higher values compared to the helium flow (by 1.16 times; *p* < 0.05).

In general, these results indicate a significant and unequal reaction of the biological system (rat organism) to external treatment with helium and argon streams.

## 4. Discussion

Interestingly, there are relatively few studies to date aimed at assessing the metabolic effects of noble gases. In the literature, it is believed that inert gases [4, 16–18], which do not have the ability to react with other compounds, cause only minimal local effects, including only short-term shifts in the functioning of the microcirculatory bed [19]. On the contrary, helium- and argon-induced changes in metabolic processes are described only in isolated studies [16, 17, 20–22]. Consequently, the available data require significant expansion and additions.

The experiments carried out to evaluate the systemic effects of inert gases (using the example of helium and argon) allowed us to establish that the gases in question contribute to the formation of a special reaction of oxidative metabolism [13, 16–18, 20–25]. In particular, the use of helium flux is a «milder» agent, only moderately but significantly enhancing free radical oxidation, but to a predominant extent increasing the antioxidant potential [12, 13, 19, 21, 24]. This is due to adaptive stimulation of the catalytic activity of the main antioxidant enzymes–catalase and superoxide dismutase; however, their induction does not go beyond physiological limits [13].

On the contrary, the impact of argon flow should be recognized as more “severe.” This factor provides stimulation of free radical processes, only partially limited by the antioxidant system [14, 25, 26]. This is evidenced by a significant increase in the activity of catalase and superoxide dismutase.

In our opinion, it is logical to consider these shifts from the standpoint of the participation of a special intracellular “molecular sensor” [14]. Our previous publications present a set of molecular processes that are extremely sensitive to the action of various physical and chemical factors. This “molecular sensor” is universal but nonspecific and is based on free radical oxidation reactions involving reactive oxygen and nitrogen species [14]. At the same time, the spectra of the bioradicals involved, their concentrations and the rate of formation can, according to our concept, determine the specificity features of the molecular, and then, the cellular, tissue, and organism response to the action of an external physical and chemical factor. In addition, these parameters determine the adaptability (physiology) or disadaptivity (formation of prepathology or pathology, up to the development of oxidative stress) of the reaction.

**Figure 8:**
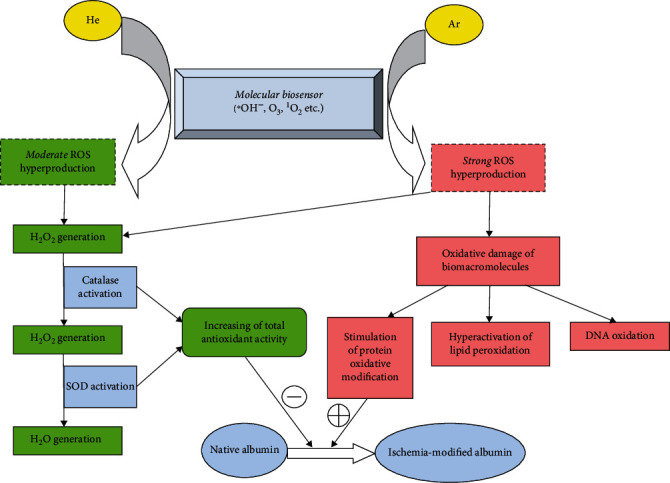
Possible molecular mechanism of the biological effects of helium and argon (*adaptive* pathway indicates in *green*; *disadaptive* pathway indicates in *red*; He: helium; Ar: argon; ROS: reactive oxygen species; SOD: superoxide dismutase; ^∗^OH^−^: hydroxyl ion; O_3_: ozone; ^1^O_2_: singlet oxygen; H_2_O_2_:hydrogen peroxide; DNA: deoxyribonucleic acid).

From the above positions, the treatment of animals with helium and argon streams can also be perceived by this “molecular sensor” (Figure 8). The results of this study allowed us to establish the potential antioxidant effect of the helium stream, mainly realized due to the activation of the catalytic properties of the enzymatic link of the antioxidant system of rat blood plasma (primarily catalase and superoxide dismutase). Such a response, according to the literature, can be caused by the generation of a moderate amount of ROS and nitrogen with the predominance of hydrogen peroxide [13]. This metabolite induces catalase stimulation with transformation into a superoxide radical utilized by the corresponding enzyme [27]. These processes provide adaptive stimulation of the antioxidant system, which allows us to consider them as a physiological way of responding to the action of helium.

We assume a different mechanism in relation to the treatment of animals with argon flow. In this case, as with the use of helium, the signal will be perceived by a universal “molecular sensor,” also leading to the generation of ROS and reactive nitrogen species [14]. On the one hand, these bioradicals, including hydrogen peroxide, will activate antioxidant enzymes, activating the physiological response pathway [9]. At the same time, the revealed features of shifts in oxidative metabolism, including not only stimulation of the antioxidant system but also pronounced induction of free radical oxidation, indicate the connection of additional molecular cascades. Apparently, under the action of the argon flow, an “excess” of reactive oxygen and nitrogen forms is formed, some of which initiates the physiological pathway, and the rest are spent on oxidative modification of biomacromolecules [21]. This process affects lipid peroxidation, oxidative modification of proteins and nucleic acids, and the induction of carbohydrate metabolism [4, 13, 28–30]. We verified the effect on these reactions by the dynamics of the concentration of ischemically modified albumin, which decreased to a greater extent when using helium compared to argon. The selectivity of the revealed effect is due to the fact that helium mainly activates the physiological pathway of the “molecular sensor,” providing stimulation of the antioxidant system that prevents oxidative modification of albumin. When treating rats with argon flow, this process competes with the oxidizing effect of the “excess” of the formed reactive oxygen and nitrogen forms, limiting the protective effect of the antioxidant system with respect to the oxidative transformation of albumin. This leads to a less pronounced decrease in the concentration of ischemically modified albumin in the case of the use of argon, compared with that observed with the use of helium.

Thus, the conducted studies made it possible to verify the specificity of the response of the oxidative metabolism of blood plasma to the use of inert gases, depending on their type. These features may be due to the connection of various molecular response pathways to the action of helium and argon; however, it should be emphasized that these shifts in any case cannot be interpreted as pathological without evaluating cellular and tissue reactions.

The metabolic effects of noble gases revealed because of the study are essential for deciphering the mechanisms of their therapeutic use [11]. In particular, the effectiveness of the use of argon, helium, and other inert gases in the correction of ischemic and reperfusion disorders in the corresponding models in rabbits and rats of various lines has been experimentally shown. The use of helium, argon, xenon, and neon (most often as part of a helium-oxygen gas mixture) contributed to the reduction of the infarction zone [3, 6, 8]. In addition, argon inhalation made it possible to limit the rate of decline in left ventricular function in experimental infarction [31]. There is also evidence of neuroprotective [32] and renoprotective activity [33] of several noble gases. In all the above effects, the directed correction of oxidative metabolism will contribute to the elimination of ROS-induced damage to cells and tissues.

## 5. Conclusions

The results of the conducted studies indicate that the external use of inert gas streams (helium and argon) causes the formation of significant shifts in the oxidative metabolism of rat blood, and these changes are gas-specific. It has been established that both of the considered effects provide stimulation of antioxidant systems, but its mechanism is not the same. Thus, the treatment of animals with helium, while maintaining the intensity of free radical processes at the control level, significantly increases the overall antioxidant activity of blood plasma and significantly reduces the concentration of malonic dialdehyde compared with the use of argon flow. This may be due to the involvement of different intracellular signaling pathways (adaptive or disadaptive) under the influence of the studied noble gases.

## Data Availability

Data supporting this research article are available from the corresponding author or first author on reasonable request.
